# Dr. Dewees's Examination of Dr. Osborn's Opinion of the Physical Necessity of Pain and Difficulty in Human Parturition

**Published:** 1807-11

**Authors:** 


Dr. Dewees's *Examination of* Dr. Osborn's *Opinion of the Physical Necessity of Pain and Difficulty in Human Parturition.*

DR. OSBORN, in the introduction to his essay on laborious parturition, has endeavoured to prove, that pain and difficulty are natural to woman in parturition. He conceives, " that in sorrow shalt thou bring forth children," was a curse pronounced by God against man, and that it was his intention it should be fulfilled and continued as long as the world endured. That this curse was felt and perpetuated, by the erect form which he gave man; while the horizontal one of the subordinate quadruped exempted it from these evils; he also supposes, " the peculiar advantages of position so different from each other, can no more exist in the same creature, than the strength of the dray-horse and the fleetness of the racer, can be united in the same animal: as these depend on qualities incompatable with each other, and which cannot exist together in the same subject, so those depend on circumstancess of structure, or physical laws, equally incompatible and inconsistent."

From this it would appear, first, that God intended that woman should bring forth with pain and difficulty; and secondly, that this intention was answered by a physical peculiarity, *that is*, an erect form. From these positions I must dissent. God, in giving the erect form to man, could not mean it should serve as a balance, to the disadvantages resulting from it to the female; he intended man for the most perfect, and the most powerful animal; he gave him faculties, capacities, and appetites, different from all others; he gave him the erect form as the most dignified, and the best calculated to display and improve those transcendant advantages. It would indeed be limiting the power of the Deity, to suppose, that a mechanism so elaborate, and so perfect perfect as that of man, was necessary to effect a curse (as Dr. O. believes it) so limited. Had this been the intention of God, why should not the male participate in its disadvantages? or, in other words, why should the female alone incur the penalty? since the Doctor himself admits, that, except for this, the erect form is a mark of pre-eminence, and a blessing.

Besides, the physical necessity of pain and difficulty is by no means proved, by the text he has brought forward to demonstrate it. For, "in sorrow shalt thou bring forth children," does not necessarily imply, they shall be brought forth with pain and difficulty; for sorrow is, in no one instance in the holy writings, made synonimous with pain or difficulty; in no one instance is it made to signify corporeal sensation: on the contrary, it is invariably used to express a certain painful state of mind. I therefore believe, it was only intended to express the anxiety every woman feels for her own safety, and for that of her infant, at the interesting moment of her becoming a mother. This state of mind is inseparable from the pregnant woman; the joyless savage on the banks of the Oroonoko, is not more exempt from it, than the enervated female of civilized society. When she reflects on what uncertain tenure life is held, that one half, or more, of the human race is doomed to certain death before they arrive at maturity; the variety of accidents, as well physical as moral, the heir of man is exposed to, she sorrows, and "in sorrow" she brings forth.

This I conceive to be the true meaning of the text quoted by Dr. O.; for were it otherwise, and made to signify pain and difficulty, it would necessarily imply punishment; this punishment ought universally to obtain, agreeably to the intention with which it is said to have been inflicted: but this is not the case; we therefore cannot suppose it was intended as a punishment. On the contrary, it is more than probable that pain and difficulty are artificial, and are the consequences merely of civilization and refinement. For the human constitution, when not under the influence of these causes, will, *cæteris paribus*, be found capable of meeting and overcoming, without any difficulty, the ordinary changes produced by gestation and delivery. Of this, abundant proofs might be given; for the female savage, wherever found, whether under the scorching heat of an African sun, or beneath the rigorous sky of the unfriendly Labrador, brings forth her young, without the assistance of accoucheur or midwife; but the reverse reverse of this almost universally obtains among the females of the civilized world; these differences are most probably occasioned by the changes produced on the human constitution, by civilization and refinement.

The mischiefs derived from the sources just mentioned, are found to consist in the disposition to, or existence of diseases, either general or local, or both; in those which may affect the system in general, or those which may exist in the uterus or pelvis in particular; in the introduction and continuance of certain pernicious customs, habits, or modes of life, thereby inducing a preternatural degree of inability, sensibility, laxity, or rigidity, and hence the physical necessity of pain and difficulty in parturition, among the greater part of women in a state of civilization and refinement. The difference then, in the opinions of Dr. O. and myself, consists in, what he supposes natural and unavoidable, I believe artificial and in part remediable.

I will now examine the Doctor's arguments in favour of this natural physical necessity; and if their futility or inconclusiveness can clearly be shewn, I trust my point will be established, without the necessity of much positive reasoning.

Dr. O. thinks it as incompatible to unite the advantages of positions, so different as those of man and the quadruped, as it would be to unite the strength of the draft-horse with the fleetness of the racer; yet it is well known that many women being forth children without pain, consequently the horizontal position of the brute is not exclusively the only one, in which a fœtus may be born without it.

Dr. O. having laid down his favourite positions—namely, that pain and difficulty were intended by the Deity as concomitants on human parturition; and secondly, that these were effected, by the erect form of man; goes on to consider how this is brought about; "to understand" says he, "how the erect position of body necessarily operates, in making natural labour in woman more painful, tedious, and difficult than in the quadruped; it is sufficient to observe, that in such a situation, there is the general and powerful influence of gravitation to counteract, in a certain degree, during the whole period, but in a much greater degree towards the conclusion of utero-gestation; for as gestation advances, the ability in the soft parts to resist the influence of gravity, regularly decreases: and thus if not prevented, premature labour would be the inevitable consequence."

"Com-

" Completely to guard against this accident, which is of the last importance to the existence of mankind, nature has taken particular pains, and attended to a variety of circumstances in the structure of the bodies, both of mother and child, which, while they effectually answer the purposes intended, unavoidably create those very obstacles which delay and impede delivery. The most natural of these circumstances it may be proper to describe."

" First, then, that irregular cylindrical cavity in the skeleton, called the pelvis, through which the fœtus in all animals must pass, is so placed in the human body, that its axis is very different from the axis of the trunk, and of course, not perpendicular to the horizon, nor can any thing passing through it, be within the immediate influence of gravity ; at the same time, the axis of the pelvis is very remote from, if not directly opposite to, the axis both of the vagina and os externum, through which the fœtus must pass."

These positions are certainly true ; but what do they prove? Certainly not, that pain and difficulty are inevitable to the animal so circumstanced, in bringing forth its young; but merely, that the different axis of the trunk, pelvis and vagina, are not parallel : but this makes nothing for the point, since it by no means follows, that women, to be exempt from pain and difficulty in labour, must have these axes correspond. This indeed would be a disadvantage, agreeably to Dr. O's own confession: for he alleges, that were the influence of gravitation not taken off, premature labour would be the consequence ; but this end is effectually answered, by giving this variety to the axis of the parts concerned in parturition: and this, without necessarily being the cause of pain and difficulty, since we see women bring children without them ; for the most perfect correspondence takes place successively, between the different axes as the labour advances.

" Secondly, upon the same principle," says Dr. O· " and with the same view, nature has been obliged to vary, nicely and minutely, both the form and the capacity of the pelvis, making it wide in one part, narrow in another, concave and deep behind, straight and shallow before, and with sides that converge to a considerable degree."

The arrangement here spoken of, though correct, I do not by any means conceive was intended for the purposes Dr. O. supposes, (namely, pain and difficulty) any more than the one just spoken of, since the same argument must necessarily hold good in both cases, viz. women are delivered without pain, who possess all this variety in their pelves.

"Thirdly, the upper and lower apertures of the pelvis do not all correspond in their shape, and have directly opposite diameters. The superior aperture or brim of the pelvis, where the child enters, is oviform, with the long diameter extending from side to side. The inferior aperture, through which the child is to pass out, is so irregular as not to admit of a comparison, or illustration, from any known form, but is certainly shorter from one side to the other, than from the fore to the hind part; and that, in nearly the same proportion as it was longer above: thus the two apertures have directly opposite diameters."

The construction of the pelvis spoken of does not necessarily produce pain and difficulty in parturition, unless from a wrong position of the fœtus, since the diameters of the head are made to correspond with those of the pelvis; and this arrangement is essential, for the reasons just mentioned; since by it, two great objects are accomplished; first, the woman is not subjected to abortions when the uterus is impregnated, nor to its prolapsus, when empty; secondly, a resting-place is furnished to the fœtus by the hard and soft parts of the mother, whereby she is enabled to carry her burden to the most remote period of gestation, without any very great inconvenience to herself, or danger to the child.

"Fourthly, pursuing the same intention, nature has made the volume of the child's head such, compared with the cavity of the pelvis, that it cannot enter by its own weight, but requires the powerful and repeated contractions of the uterus and abdominal muscles, and even then, the head must be of a particular form, and in a particular direction; that in the passage, both these necessarily undergo a material change from compression, that the shape of the head may be, all through, adapted to the pelvis; and thus it must come out with an altered form, and in a different direction."

This argument in favour of the physical necessity of pain, &c. can have but little weight, since it amounts to no more than what we have already granted, and are again willing to concede—that, for the head to pass through the pelvis, it must have its large and small diameters correspond with those of the pelvis. This most frequently obtains, and with such uniformity, that the head might very frequently be still larger, without augmenting or producing difficulty.

culty. We cannot here withhold our admiration at the wonderful resources and simplicity of nature in this construction; that, while it affords a security from certain accidents to the animal to be born, it at the same time gives a freedom from evils to the mother, that would be irremediable; and all this is effected, unfortunately for the Doctor's argument, without necessarily producing pain and difficulty.

The general position of the child's head in utero, is such, that its longest diameter traverses the pelvis rather diagonally; by this arrangement, portions of the greatest diameter of the head are constantly presented to portions of the smallest diameter of the superior strait, which prevents the head from engaging in it by its gravity; and consequently, exempts the mother from some serious inconveniences by this provision.

In brutes there is no necessity for this arrangement; as their pelves are nearly, or quite, horizontal, the axes of their uteri are not exactly paralled with the axes of the pelvis and vagina; since, the weight of the contents of their uteri carries them below their plane, and consequently, requires as much assistance from the contractions of their wombs to adapt the different axes, as it does in the human subject; but this contraction or effort in the brute is not attended with pain; we cannot therefore conclude it necessarily productive of it in women. Besides, nearly similar changes must take place in the birth of the inferior animals, as in the human female; since it cannot be supposed that a similar conformation was given them, for a different purpose; for instance, in all the pelves of brutes we examine, we find an inflexion of the lumbar column; a difference in the diameters of the first strait; a corresponding difference in the second; a greater or lesser excavation of the sacrum, and a looking in of the coccyx. The heads of animals also, bear a very strong general analogy to the human; such as a difference in diameters, to answer the different diameters of the pelvis; sutures and fontanelles, in order that the head may conform to the changes, necessarily imposed on it by the contractions of the uterus in its passage through the pelvis. In them also, as in the human subject, do difficulties occur, from a wrong situation of the head, or from an unfortunate presentation of some other part; in them also do the same consequences result when this happens,—pain and difficulty.

"To add to the more effectual support of the gravid uterus during gestation, all the soft parts concerned in labour are of a firm and rigid texture, dilating at all times with considerable difficulty, to such a degree as to permit the passage of the child through them without laceration, or other injury. It is obvious that these circumstances must render the act of child-bearing, slow, difficult, and painful."

These last assertions of Dr. O. cannot be admitted, since every day's experience contradicts them. It is a well known fact to every practitioner, that in very many instances, the mouth of the uterus and other soft parts concerned in parturition, yield as kindly with the first child as they do with subsequent ones; and that, sometimes, there is with the first an unwillingness in these parts to yield, that never manifests itself again: besides, these changes take place sometimes so rapidly, and so silently, that the woman is often surprised alone, by the birth of her child. Pain is certainly not necessary to relaxation, nor does relaxation produce pain. Pain may continue violently and for a long time without producing the smallest relaxation; indeed, it seems unfavourable to it, since it implies more or less disease, or at least, a greater or lesser departure from the ordinary process of nature. Besides, did we admit, that the rigidity of the soft parts and their unwillingness to yield, must necessarily make "the act of child-bearing, slow, difficult, and painful," with women in a certain state of civilization and refinement, it does not follow as a consequence, that this must universally obtain!

Again, it may safely be asserted, that pain and difficulty in those who may experience them, do not in general depend on rigidity, which Dr. O. considers as a law of nature, and one that firmly establishes his theory; for we find them frequently taking place after the most perfect relaxation; consequently, they cannot be considered as essential to this effect, since the end is answered; and, moreover, relaxation only takes place in the absence of pain, for pain is produced by contraction.

No, it is to the changes already hinted at, as produced by civilization and refinement, that we are, for the most part, to attribute the pain and difficulty of human parturition, and not to the peculiar structure of the body. These evils rarely occur among savages, or among those who have not been injured by disease, or perverted by custom. So little trouble has the Squaw of this country in her labour, that it never interrupts any project or enterprise she or her husband may have in view; if taken in labour when marching marching with him, she retires behind a bush, delivers herself, and quickly again rejoins him.

Mr. Swinburne tells us* that the women of Calabria bring forth their offspring almost without a groan; and that it has become proverbial, "that a Calabrian maid-servant prefers the labour of child-birth to that of a wash."† M. Brydone‡ also informs us, that among the Sicilian women, labour is attended with so little pain or danger, that they appear perfectly well the day after delivery. Many more instances of the like kind might be mentioned from authors, which seem clearly to prove, that this operation was intended by the Deity, to be performed with ease and safety.

How different is this from what we observe among women, who have been perverted by civilization and refinement! what miseries have not our boasted improvements inflicted! The curse that has fallen upon us has been from luxury, instead of being the fruit of the disobedience of our first parents.

( To be continued. )

* Travels in the Two Sicilies, page 287. † Ibid

‡ Tour through Sicily and Malta, vol 2, letter 22.

